# Effects of task-oriented circuit class training on walking ability after stroke: a meta-analysis

**DOI:** 10.3389/fneur.2026.1724575

**Published:** 2026-03-04

**Authors:** Lin Chen, Xiuli Wei, Shenshen Zheng

**Affiliations:** 1 Department of Rehabilitation Medicine, Renmin Hospital, Hubei University of Medicine, Shiyan, Hubei, China; 2 Department of Rehabilitation Medicine, Taihe Hospital, Hubei University of Medicine, Shiyan, Hubei, China

**Keywords:** circuit class training, meta-analysis, rehabilitation, stroke, walking ability

## Abstract

**Objective:**

Task-oriented circuit class training (CCT) is increasingly used in the rehabilitation of post-stroke gait dysfunction; however, current research findings remain inconsistent. This study aimed to further investigate its therapeutic efficacy.

**Methods:**

Randomized controlled trials (RCTs) evaluating task-oriented CCT in stroke patients were identified through a systematic search of PubMed, the Cochrane Library, and EMBASE, covering the period from database inception to February 25, 2025. Studies were included if they assessed lower limb functional outcomes. Only publications in English were considered. Two independent reviewers conducted literature screening, data extraction, and risk-of-bias assessment. Meta-analysis was performed using Review Manager 5.4 and Stata 18.0 software.

**Results:**

Twelve RCTs comprising 652 patients were included. Meta-analysis demonstrated significant, homogeneous effect sizes in favor of task-oriented CCT for the 6-min walk test (mean difference (MD) = 57.88, 95% CI 33.43 to 82.32, *p* < 0.00001), the Timed Up-and-Go test (MD = −1.74, 95% CI: −2.92 to −0.57, *p* = 0.004), and gait speed (MD = 0.13, 95% CI: 0.06 to 0.20, *p* = 0.0002). Subgroup analysis indicated that in patients within 3 months post-stroke, implementing task-oriented CCT with increased training frequency (≥3 times/week) was associated with improvements in 6-min walk distance and gait speed. In patients more than 3 months post-stroke, higher training frequency (≥3 times/week) or longer session duration (≥1 h) was linked to greater gains in 6-min walk distance and Timed Up-and-Go test (TUG) performance.

**Conclusion:**

These findings support the beneficial effects of task-oriented CCT in improving walking ability after stroke. Future large-scale, multicenter RCTs are warranted to compare the effects of varying training components, including content, intensity, single-session duration, and intervention timing, on post-stroke walking function.

**Systematic review registration:**

https://www.crd.york.ac.uk/PROSPERO/home, identifier CRD420250652683.

## 1 Introduction

The most recent epidemiological studies on stroke indicate that among non-communicable diseases (NCDs), stroke ranks as the third-leading cause of death and disability worldwide, measured by disability-adjusted life years lost (DALYs), exceeding 160 million DALYs annually (1). The World Stroke Organization/Lancet Neurology Stroke Commission projects that by 2050, the global number of stroke survivors will surpass 200 million, with annual DALYs approaching 300 million (2). Stroke is the leading cause of long-term neurological disability worldwide (3).

About 80% of stroke survivors experience walking problems (4), and achieving safe, independent, effective, and efficient walking is a top priority for improving their quality of life (5). About 75% of patients consider the capacity to engage in community activities is importance (6). Nevertheless, about one-quarter of stroke survivors cannot walk independently within 3 months post-stroke (7). Persistent gait abnormalities, including reduced gait speed and endurance, combined with not enough rehabilitation intensity, significantly limit ability recovery after stroke. Rehabilitation training plays a crucial role in improving limb ability, particularly about lower limb rehabilitation (8).

Task-oriented circuit class training (CCT) is a task-oriented intervention in which participants receive physical rehabilitation treatment in a group setting, typically with at least two participants per therapist (9). The program emphasizes repetitive practice of numerous function- and activity-related tasks under the supervision of a physical therapist (10). Based on current knowledge in neuroscience and stroke research, effective recovery requires task-specific practice, sufficient repetition, and high-intensity training sessions (11, 12). Kelly et al. (13) developed a task-oriented circuit training program termed intermittent functional training (IFT) and compared its initial effects with those of constant-load endurance treadmill training (CET). Their findings indicated that task-oriented circuit training enables individuals with chronic hemiparetic stroke to sustain comparable levels of aerobic exercise intensity during training relative to conventional force-measured exercise protocols. Similar to individual training, task-oriented CCT is equally effective in improving balance and ambulation (14). In a large randomized controlled trial (RCT) involving 250 patients, the intervention group underwent 12 weeks of task-oriented CCT, while the control group underwent 12 weeks conventional physical therapy. At 12 weeks, the intervention group demonstrated significantly greater improvements in gait speed and walking distance than the control group (15). In addition, growing evidence suggests that intensive, task-oriented practice can produce greater improvement in walking competency than usual practice (10, 16, 17).

A 2017 systematic review published in the Cochrane Database of Systematic Reviews analyzed 17 randomized controlled trials encompassing 1,297 participants, comparing circuit training rehabilitation with conventional care or sham interventions. The review provided moderate-quality evidence that circuit training is more effective than alternative approaches in improving walking distance, independent ambulation, gait speed, and, to some extent, balance confidence and postural stability. Notably, benefits were observed regardless of time since stroke onset—patients more than 1 year post-stroke experienced improvements comparable to those within the first year, indicating that functional gains may extend well beyond the early recovery period (9). However, the review did not evaluate the impact of stroke type, severity, specific circuit training protocols, or training intensity on outcomes. Given the growing global burden of stroke and recent advances in neurorehabilitation, a comprehensive update of the current evidence is warranted to address these unresolved issues. In this study, we conducted a meta-analysis to assess the efficacy and safety of task-oriented CCT on lower limb ability post-stroke.

## 2 Methods

### 2.1 Study registration

This review adhered to the Preferred Reporting Items for Systematic Reviews and Meta-Analyses (PRISMA) and Cochrane Collaboration guidelines for conducting and reporting systematic reviews and network meta-analyses (NMAs) (Appendix 1) (18). The protocol for this research was previously registered with PROSPERO (registration number CRD420250652683).

### 2.2 Search strategy

A systematic literature search was conducted in PubMed, Embase, and the Cochrane Central Register of Controlled Trials from inception to February 25, 2025, focusing on group circuit training for lower limb rehabilitation in stroke patients. The following medical subject headings (MeSH) and keywords were used: “stroke,” “cerebrovascular accident,” “cerebrovascular disease,” “hemiplegia,” “paresis,” “brain injuries,” “circulatory training,” “circulatory courses,” “exercise,” “task-oriented,” “gait,” “walking,” “lower limb,” and randomized controlled trials (“RCTs” OR “trial” OR “trials”). The detailed search strategy is provided in Appendix 2. Additionally, references from review articles, empirical studies, and conference abstracts were screened. References of the retrieved articles were also reviewed to identify other relevant trials meeting the inclusion criteria. All searches were limited to RCTs conducted in humans.

### 2.3 Inclusion and exclusion criteria

Studies were eligible for inclusion if they met the following criteria: (1) Study subjects: adult stroke patients (≥18 years) as defined by the World Health Organization, with lower limb functional impairments, capable of walking at least 10 meters with or without assistive devices (functional ambulation category ≥2); randomized clinical trials must have evaluated task-oriented interventions targeting the lower extremities in a group setting, with at least one outcome related to gait. (2) Types of studies: RCTs published in English with extractable outcome data. (3) Intervention measures: the experimental group received task-oriented CCT focusing on the lower limbs with at least two participants per group, while the control group received one-to-one traditional training or no treatment or other forms of treatment. Studies were excluded if: (1) the intervention included additional therapeutic approaches, such as motor imagery or mirror therapy; (2) the outcomes were unrelated to lower limb function or data were incomplete; (3) the publication was a review, conference proceeding, experience report, or case study; or (4) the study was a duplicate publication.

### 2.4 Study selection and data extraction

Two independent reviewers (Xiuli Wei and Lin Chen), blinded to study details, used Endnote software to perform systematic screening, including title and abstract review followed by full-text assessment. Discrepancies identified during the title/abstract screening were advanced to full-text review, and disagreements during full-text review were resolved through consensus. Extracted data included author, publication year, allocation concealment method, random sequence generation technique, blinding procedures, sample sizes for both experimental and control groups, intervention and control details, study characteristics, follow-up duration, and functional outcome scores. The Cochrane Risk of Bias Tool version 2.0 (19) was employed to assess study quality, evaluating random sequence generation, allocation concealment, blinding of participants/personnel, blinding of outcome assessment, incomplete outcome data, selective reporting, and other potential biases. Studies were categorized as having low, moderate, or high risk of bias based on the Cochrane Risk of Bias 2.0 guidance. The primary outcomes were the 6-min walking test distance and the Timed Up-and-Go test score. Secondary outcomes included the Berg Balance Scale, gait speed, and the Modified Barthel Index. Data were extracted for measurements taken before, during, and after treatment.

### 2.5 Data analysis

#### 2.5.1 Literature quality assessment

Review Manager 5.4 was used for conventional meta-analysis, and forest plots were generated. All outcome measures were continuous variables, effect estimates were presented as mean differences (MD) and 95% confidence intervals (CIs). Statistical heterogeneity was assessed using the chi-squared test and *I*^2^ statistic. Heterogeneity across studies with different outcome measures was quantitatively assessed using the *I*^2^ statistic. An *I*^2^ ≤ 50% and a *p* ≥ 0.1 were interpreted as indicating low heterogeneity, in which case a fixed-effect model was applied for analysis; otherwise, a random-effects model was employed. Subgroup and sensitivity analyses were conducted to explore potential sources of heterogeneity.

#### 2.5.2 Sensitivity analysis

Sensitivity analysis was further used to evaluate the influence of individual studies on the overall effect estimate and to assess the robustness of the pooled effect size (20, 21).

#### 2.5.3 Publication bias

To evaluate potential publication bias in the study outcomes, we generated funnel plots using Stata 18.0 software. Visual inspection of funnel plot symmetry was used to judge the presence of publication bias: approximate symmetry suggested no substantial publication bias, whereas asymmetry indicated a possible risk of bias (22). Due to expected variability in study design, outcome measures, and sample sizes, a random-effects model was applied. Analysis of publication bias in the 6-min walking test score in this study.

#### 2.5.4 Subgroup analysis

To investigate the effects of task oriented CCT on outcome indicators and evaluate its long-term efficacy, subgroup analysis was conducted on disease duration, weekly training frequency, single training time, and follow-up time, and these results can be obtained from the study.

## 3 Results

### 3.1 Results of studies selection

**Figure 1:**
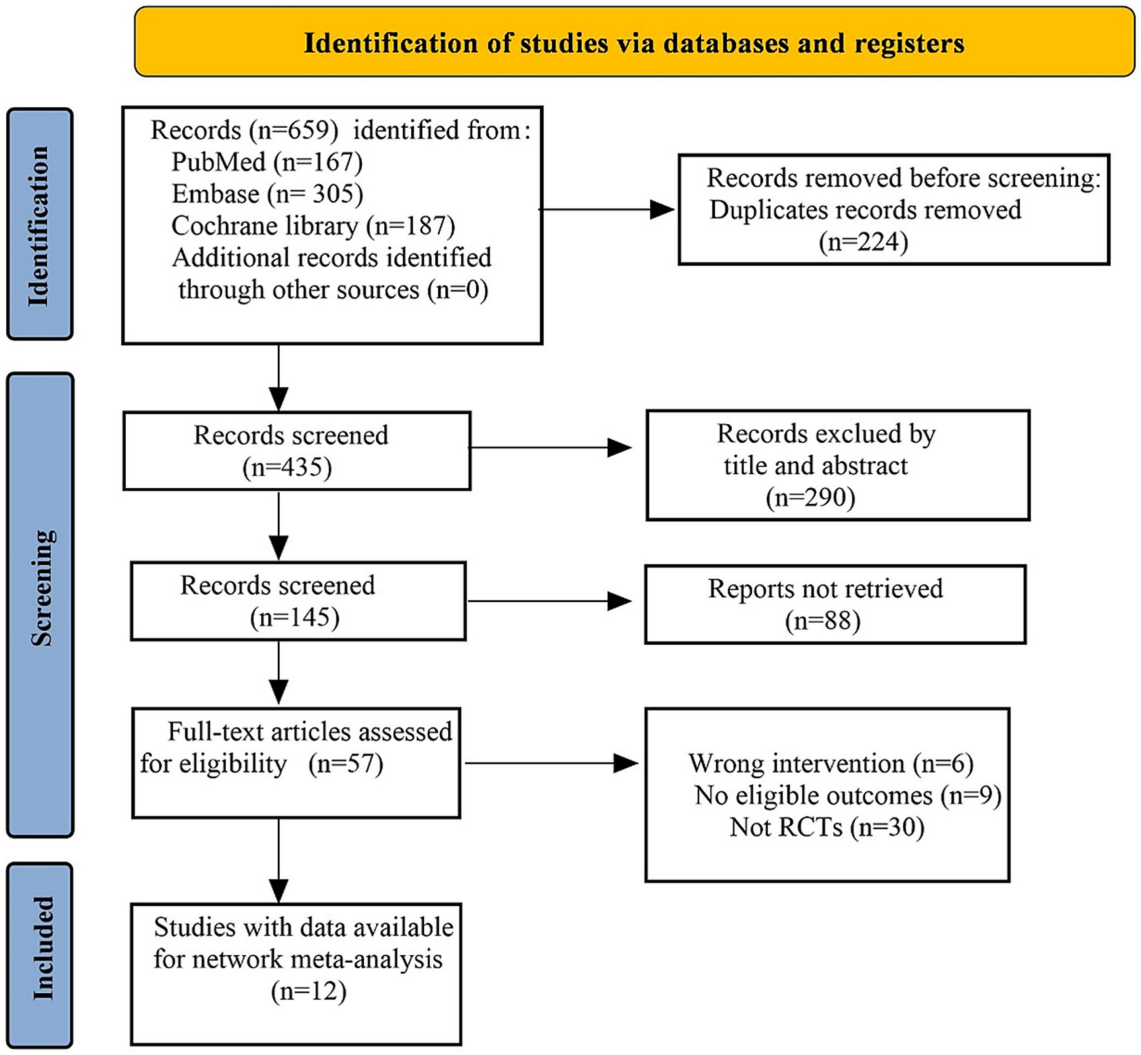
Flow diagram of study selection.

A total of 659 studies were initially identified. After removing duplicate publications, 435 studies remained for further screening. Following evaluation of titles and abstracts, an additional 290 studies that failed to meet the inclusion criteria were excluded. Among the remaining 145 studies, conference abstracts and clinical trial registrations were further excluded. A total of 57 full texts were evaluated for eligibility. Among them, 30 were excluded as non-RCTs, 6 due to intervention measures not meeting the pre-defined criteria, and 9 because of unidentifiable or incomplete outcome data. Ultimately, 12 RCTs were included in the final analysis (14–17, 23–30) (Figure 1).

### 3.2 Characteristics of included studies

The 12 included studies encompassed a total of 652 stroke patients, of whom 390 were male and 242 were female (a study (17) did not report the male-to-female ratio). Regarding age, 11 studies provided mean values and standard deviations, while one lacked precise data (30). A total of 326 cases were assigned to the experimental group and 326 to the control group for statistical analysis. The mean ± standard deviation of age was 55.3 ± 11.87 years in the experimental group and 55.79 ± 11.47 years in the control group, with no statistically significant difference between groups. Two articles included three control groups (18, 29), whereas the remaining 10 each had two control groups (15, 17, 24–28, 30, 31). Regarding stroke type, there were 121 cases of hemorrhagic stroke and 362 cases of ischemic infarction. Four studies did not specify stroke subtype (17, 24, 25, 28). Concerning hemiplegia laterality, 179 cases involved left-sided paralysis and 173 involved right-sided paralysis; however, three studies did not report this information (15, 17, 25). In terms of post-stroke intervention timing, five articles described interventions initiated within 3 months post-stroke (14, 27–30), while six reported interventions starting at 3 months or later (15–17, 24–26); one study did not specify the timing (23). Follow-up data were absent in six articles (17, 23, 25–27, 30) and available in the remaining six (14–16, 24, 28, 29), with a total of 434 cases followed up and 69 dropouts recorded. Among the studies with follow-up, two reported outcomes at 1–2 months (24, 30), and four provided 3-month follow-up data (15, 16, 28, 29). Outcome measures included the 6-min walk test (reported in 9 studies) (15, 16, 24–30), the BBS (5 studies) (14, 25–28), the Modified Barthel Index (MBI) (4 studies) (15, 27, 29, 30), the TUG test (8 studies) (14, 15, 23–26, 28, 29), and gait speed (7 studies) (15–17, 24, 28–30) recorded a comfortable walking speed of 10 meters without external support. None of the included studies reported on limb spasticity or adverse treatment reactions. Baseline activity levels showed no significant differences between the experimental and control groups across all studies. Detailed information is provided in Tables 1, 2.

**Table 1:** Baseline characteristics of the studies.

No.	First author, year	Treatment group						Control group							
		*N*	(M/F)	Age (years) (mean ± SD)	Stroke side (L/R)	Stroke type (H/I)	Duration (mean ± SD)	N	(M/F)	Age (years) (mean ± SD)	Stroke side (L/R)	Stroke type (H/I)	Duration (mean ± SD)	Follow-up	Outcomes
1	Dean et al. (2000) (24)	6	3/3	68.8 ± 4.7	3/3	NR	2.1 ± 0.5 y	6	4/2	64.8 ± 3.3	2/4	NR	1.7 ± 0.9 y	8 W	②④⑤
2	Verma et al. (2011) (30)	15	10/5	53.27 ± 8.5	7/8	3/12	6.07 ± 3.30 w	15	12/3	55.07 ± 6.8	8/7	4/11	6.60 ± 3.20 w	6 W	①②⑤
3	van de Port (2012) (15)	126	82/65	56 ± 10	NR	23/103	91 ± 42 d	124	80/44	58 ± 10	NR	24/100	103 ± 51 d	24 W	①②④⑤
4	Song et al. (2015) (17)	10	NR	59.28 ± 5.2	NR	NR	27.66 ± 19.35 m	10	NR	64.10 ± 8.6	NR	NR	30.70 ± 14.68 m	No	⑤
5	Renner et al. (2015) (29)	34	22/12	56 ± 10	19/15	12/22	39 ± 25 d	39	29/10	55 ± 10	17/22	8/31	32 ± 11 d	24 W	①②④⑤
6	Kim et al. (2016) (25)	15	8/7	50 ± 9.5	NR	NR	≥6 months	15	10/5	54 ± 7.1	NR	NR	≥6 months	No	②③④
7	Kim et al. (2016) (27)	10	6/4	65.6 ± 9.2	6/4	2/8	≤3 months	10	7/3	65.6 ± 9.2	5/5	2/8	≤3 months	No	①②③
8	Kim et al (2017) (26)	15	10/5	57.3 ± 12.3	8/7	9/6	3.3 ± 1.3 m	15	9/6	54.0 ± 11.8	10/5	8/7	4.4 ± 1.6 m	No	②③④
9	Knox et al. (2018) (28)	51	25/26	51 ± 15	26/25	NR	10 ± 8 w	48	28/26	48 ± 14	25/23	NR	8 ± 7 w	24 W	②③④⑤
10	Ain et al. (2018) (23)	15	8/7	52.26 ± 10.1	8/7	5/10	NR	15	8/7	54.10 ± 10.1	7/8	5/10	NR	No	④
11	Ali et al. (2020) (14)	11	7/4	50–70	4/7	4/7	≤3 months	11	6/5	50–70	7/4	2/9	≤3 months	No	③④
12	Martins et al. (2020) (16)	18	8/10	56 ± 17	8/10	NR	≥6 months	18	8/10	55 ± 13	9/9	NR	≥6 months	16 W	②⑤

M: male; F, female; d, day; m, mouth; W, week; NR, not reported; H, hemorrhage; I, ischemia. ① MBI: Modified Barthel Index; ② 6MinWT: 6-min walk test; ③ BBS: Berg Balance Scale; ④ TUG: Timed Up-and-Go test; ⑤ Gait speed.

**Table 2:** Intervention measures of the studies.

No.	Study (year of publication)	Patients per group	No. of therapists attending	Intensity (I) Progression (P)	Workstations applied in the experimental group (E), therapy applied in the control group (C)
1	Dean (2000) (24)	6	2 physiotherapists	I: 4 weeks; 3 times a week; 60 min I: 10 workstations, 5 min each P: increasing number of repetitions P: increasing complexity of workstations	E: (1) Sitting at a table and reaching in different directions for objects located beyond arm’s length to promote loading of the affected leg and activation of affected leg muscles; (2) Sit-to-stand from various chair heights to strengthen the affected leg extensor muscles and practice this task; (3) Stepping forward, backward, and sideways onto blocks of various heights to strengthen the affected leg muscles; (4) Heel lifts in standing to strengthen the affected plantar flexor muscles; (5) Standing with the base of support constrained with feet in parallel and tandem conditions reaching for objects, including down to the floor, to improve standing balance; (6) Reciprocal leg flexion and extension using the Kinetron in standing to strengthen leg muscles; (7) Standing up from a chair, walking a short distance, and returning to the chair to promote a smooth transition between the 2 tasks; (8) Walking on a treadmill; (9) Walking over various surfaces and obstacles; (10) Walking over slopes and stairs. C: Both a circuit component with subjects completing practice at a series of workstations (e g, wrist extension, supination, grasp., and release of various objects) and some exercises completed in small groups Intervention: The exercise class for the experimental group focused on strengthening the affected lower limb and practicing functional tasks involving the lower limbs, while the control group practiced upper-limb tasks. Both experimental and control groups participated in exercise classes three times a week for 4 weeks.
2	Verma et al. (2011) (30)	≤4	1 physiotherapist 1 occupational therapist	I: 2 weeks; 7 times a week; 40 min P: increase the duration P: increase the frequency	E:(1) Balance control; (2) Stair walking; (3) Turning; (4) Transfers; (5) Speed walking. C: Subjects in the control group participated in the conventional poststroke lower extremity rehabilitation program based on the Bobath’s neuro developmental technique. Intervention: The experimental group received 15 min of MI followed by 25 min of TOCCT for a total of 40 min, 7 days per week for 2 weeks. The control group program was matched for duration, number, and frequency of the sessions with the experimental group program.
3	van de Port (2012) (15)	2	1 physiotherapist	I: 12 weeks; 2 times a week; 90 min I: 8 workstations, 3 min each P: increasing the difficulty of the task P: adding weights P: increasing the number of repetitions	E:(1) Standing and reaching; (2) Stair walking including transfer; (3) Walking and picking up various objects from the ground; (4) Kicking a ball; (5) Stepping up and down; (6) Walking course with obstacles; (7) Transfers (lying to standing and sitting); (8) Speed walking. Included four stages: warming up (5 min), circuit training (60 min), evaluation and a short break (10 min), and group game (15 min). C: Sessions designed to improve control of standing balance, physical condition, and walking competency. Intervention: The experimental group received circuit training in 90-min sessions twice a week for 12 weeks. The training included eight different workstations in a gym. The control group received usual outpatient physiotherapy.
4	Song et al. (2015) (17)	NR	2 physiotherapists	I: 4 weeks; 5 times a week; 30 min	E: (1) Sitting in chair and walking; (2) Walking over obstacles; (3) Carrying goods; (4) Turning the goods upside down; (5) Walking fast in a circle. C: Received conventional therapy for 30 min a day, five times a week Intervention: Both experimental and control groups received conventional therapy for 30 min a day, five times a week. The experimental group received TOCCT for 30 min a day, three times a week for 4 weeks.
5	Renner et al. (2015) (29)	2	1 physiotherapist or 1 sports therapists	I: 6 weeks; 5 times a week; 90 min I: 8 workstations, 3 min each P: increasing the difficulty of the task; adding weights P: increasing the number of repetitions	E: (1) Standing and reaching; (2) Stair walking including transfer; (3) Walking and picking up various objects from the ground; (4) Kicking a ball; (5) Stepping up and down; (6) Walking course with obstacles; (7) Transfers (lying to standing and sitting); (8) Speed walking. Warming up (10 min), task training (60 min), sports and games (15 min) and cooling down (5 min) C: Tailored to the deficits of the patient and aimed to improve balance, physical condition and walking competency. Intervention: The experimental group received a 90-min, structured progressive task training program, five times a week over a 6-week period. The control group received a 90-min, progressive, individually tailored task training 5 times a week over a 6-week period, offered by one of the staff physical therapists.
6	Kim et al. (2016) (25)	2–3	1 physiotherapist	I: 6 weeks; 3 times a week; 50 min I: 8 workstations, 3 min each	E:(1) Straight leg raise; (2) Kicking a ball toward the wall; (3) Sitting up and walking; (4) Obstacle walking; (5) Treadmill walking; (6) Maximal speed walking; (7) Sitting on a Swiss ball; (8) Playing a video game for 50 min; Warm-up and cool-down for 5 min were performed before and after exercise, respectively. C: The same circuits while the control group performed on individual basis. Intervention: Both groups practiced same circuits, except the experimental group, which performed these tasks in the form of groups, while the control group performed on individual basis.
7	Kim et al. (2016) (27)	≥ 2	1 physiotherapist	I: 4 weeks; 5 times a week; 90 min	E: (1) Sit to stand; (2) Standing; (3) Walking; (4) Aerobic exercise training; (5) Strengthening training. Consisted of a 5 min warm-up period, 15 min duration of complex exercises interspersed by 1 min rest, and a 5 min cool-down period. C: Participants in the control group received conventional individual physiotherapy for 30 min twice a day (total 60 min), 5 days a week for the 4 weeks. Intervention: The experimental group received a structured circuit training program performed for 90 min, 5 days a week for 4 weeks. The control group received individual physiotherapy of neurodevelopmental treatment for 60 min, 5 days a week for 4 weeks.
8	Kim et al. (2017) (26)	2–3	2 physical therapists	I: 4 weeks; 5 times a week; 50 min I: 10 workstations, 3 min each P: the level of difficulty P: complexity P: number of repetitions	E: (1) Sit to stand; (2) Stepping; (3) Tandem standing; (4) One leg standing; (5) Reaching; (6) Walking practice; (7) Obstacles reaching; (8) Reaching; (9) Slope; (10) Stairs C: Focused on task-oriented exercise, such as strengthening exercise (resistance exercise), standing balance (using varying methods), and functional activities for gait improvement. Intervention: The experimental group received performed task-oriented circuit training, and the control group received conventional physical therapy.
9	Knox et al. (2018) (28)	4–6	1 physiotherapist	I: 12 weeks; 6 times; 90 min I: 6 workstations P: increasing the complexity of the tasks	E: (1) Improving strength; (2) Balance; (3) Task performance while standing; (4) Walking; (5) An endurance walking station. C: 90-min educational session on stroke management Intervention: The experimental group received task-oriented circuit gait training, and the control group received one 90-min educational session on stroke management.
10	Ain et al. (2018) (23)	NR	1 physiotherapist	I: 6 weeks; 4 times a week; 40–50 min I: 8 workstations, 4–5 min each	E: (1) walk; (2) One leg standing; (3) One leg standing on foam; (4) Walking on different surfaces; (5) Stair climbing; (6) Standing on balance board; (7) Walking on a set pattern on floor; (8) Moving through obstacles. C: Traditional gait training exercises for four days a week with session duration 40–50 min, six weeks. Intervention: The experimental group received performed task-oriented circuit training, and the control group received conventional physical therapy.
11	Ali et al. (2020) (14)	≥ 2	1 physiotherapist	I:6 weeks; 3 times a week; 50 min I:5 workstations P: increase in difficulty in the task	E: (1) Sit to Stand training; (2) Step up forward, backwards and sideways; (3) Trunk control and rotation; (4) Reaching out in various directions collecting an object and passing on other side; C: The same circuits while the control group performed on individual basis. Intervention: Both groups practiced same circuits, except the experimental group, which performed these tasks in the form of groups, while the control group performed on individual basis.
12	Martins et al. (2020) (16)	2–6	1 physiotherapist	I: 12 weeks; 3 times a week; 60 min I: 11 workstations, 5 min each P: Increases in speed P: number of repetitions	E: (1) Reaching; (2) Grasping; (3) Manipulation of different objects; (4) Writing; (5) Pick up and transfer jars; (6) Throw and catch balls; (7) Sit-to-stand; (8) Step; (9) Heel raise activities; (10) Walking; (11) Step up onto a step. C: 40 min of static global stretching and 20 min of memory exercises, and/or health education sessions. Intervention: The experimental group received task-oriented circuit training, and the control group received conventional physical therapy, memory exercises, and education sessions.

### 3.3 Results of risk of bias

**Figure 2:**
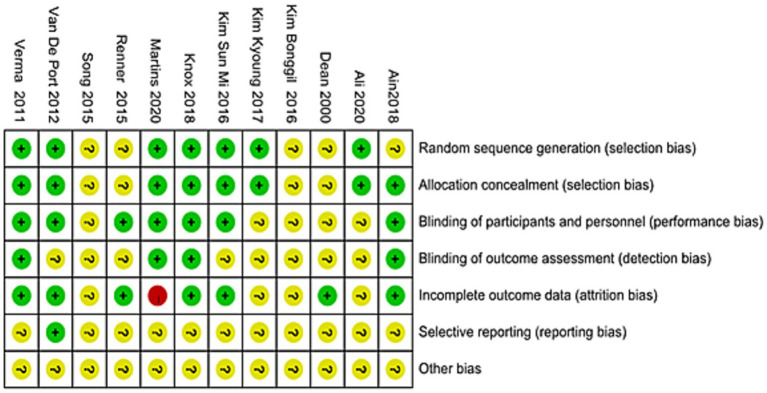
Details of assessment result of risk of bias.

Among the 12 included studies, 7 described the random sequence generation method (14–16, 26–28, 30), indicating a low risk of selection bias, the remaining studies did not report the randomization procedure (17, 23–25, 29). Seven studies reported blinding of participants (15, 16, 23, 26–28, 30), whereas the others lacked clear descriptions (14, 17, 24, 25, 29). Four studies explicitly stated that outcome assessments were free from measurement bias (16, 23, 28, 30), while the remainder did not address this aspect (14, 15, 17, 24–27, 29). One study (16) showed a substantial reduction in end-stage cases, exceeding 20% of the original sample, and was therefore classified as high risk of attrition bias. The other studies did not provide adequate follow-up data (14, 17, 25, 26), leading to an unclear risk. None of the included studies provided adequate information to assess the presence of other potential sources of bias. Further details are presented in Figure 2.

### 3.4 Outcomes

#### 3.4.1 The primary outcome

**Figure 3:**
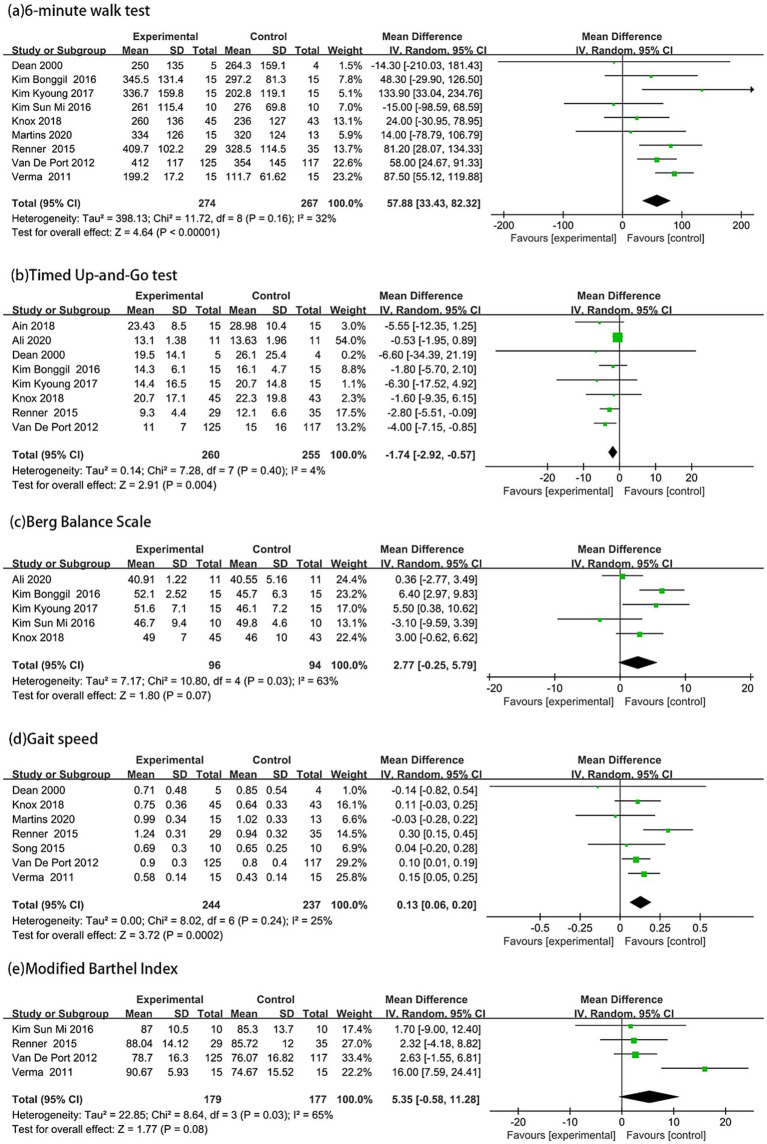
Forest plot analysis of outcome measures across the following assessments: **(a)** 6-Minute Walk Test; **(b)** Timed Up-and-Go Test; **(c)** Berg Balance Scale; **(d)** Gait Speed; and **(e)** Modified Barthel Index.

Nine articles (15, 16, 24–30) used the 6-min walk test to assess walking distance. The experimental group walked an average of 57.88 meters farther than the control group (MD = 57.88, 95% CI: 33.43 to 82.32, *p* < 0.00001, *I*^2^ = 32%) (Figure 3A). The TUG test was measured in eight studies (14, 15, 23–26, 28, 29). The experimental group was 1.74 s faster than the control group (MD = −1.74, 95% CI: −2.92 to −0.57, *p* = 0.004, *I*^2^ = 4%) (Figure 3B).

#### 3.4.2 The secondary outcome

Five articles (14, 25–28) used the BBS to assess balance ability. Showing a mean difference of 2.77 points in favor of the experimental group (MD = 2.77, 95% CI: 0.25 to 5.79, *p* = 0.07, *I*^2^ = 63%) (Figure 3C). Preferred comfortable gait speed was assessed in seven studies (15–17, 24, 28–30). With results indicating that the experimental group walked 0.13 m/s faster than controls (MD = 0.13, 95% CI: 0.06 to 0.20, *p* = 0.0002, *I*^2^ = 25%) (Figure 3D). The MBI was measured in four studies (15, 27, 29, 30). The experimental group scoring 5.35 points higher on average (MD = 5.35, 95% CI: 0.58 to 11.28, *p* = 0.08, *I*^2^ = 65%) (Figure 3E).

### 3.5 Subgroup outcomes

#### 3.5.1 Subgroup analyses of different post-stroke durations

**Figure 4:**
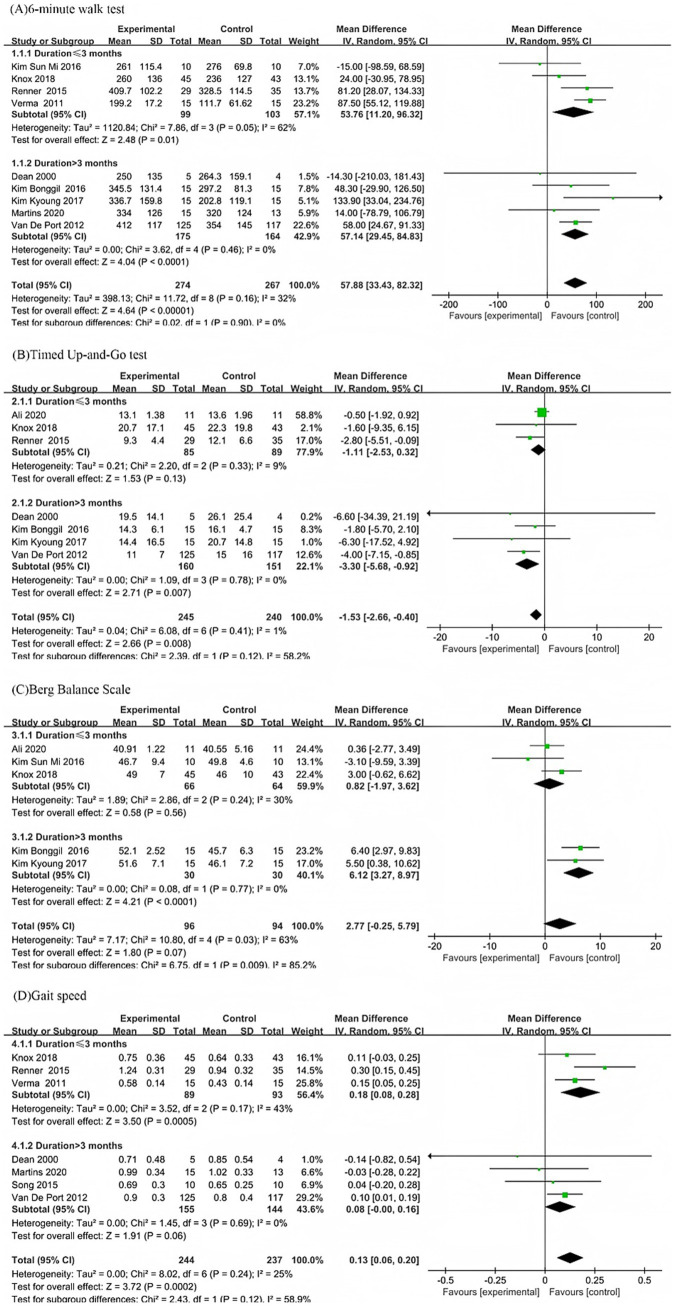
Forest plot subgroup analysis by time since stroke: **(A)** 6-Minute Walk Test; **(B)** Timed Up-and-Go Test; **(C)** Berg Balance Scale; and **(D)** Gait Speed.

The subgroup analysis based on stroke duration showed that in the 6-min walk test, the experimental group performed better than the control group in both subgroups. In the subgroup with disease duration ≤3 months, heterogeneity was relatively high (MD = 53.76, *I*^2^ = 62%), while in the subgroup with disease duration > 3 months, heterogeneity was relatively low (MD = 57.14, *I*^2^ = 0%). There was no statistically significant difference in effect size between the two subgroups (*p* = 0.90) (Figure 4A). In the TUG test, in the subgroup with disease duration ≤3 months, there was no significant difference between the experimental group and the control group (MD = −1.11, *p* = 0.13, *I*^2^ = 9%). In the subgroup with disease duration > 3 months, the experimental group was on average 3.30 s faster than the control group, with a significant difference and low heterogeneity (MD = −3.3, *p* = 0.007, *I*^2^ = 0%). There was no statistically significant difference in effect size between the two subgroups (*p* = 0.12) (Figure 4B).

In the BBS, in the subgroup with disease duration ≤3 months, there was no significant difference between the experimental group and the control group (MD = 0.82, *p* = 0.56, *I*^2^ = 30%). In the subgroup with disease duration > 3 months, the experimental group performed better than the control group (MD = 6.65, *p* < 0.0001, *I*^2^ = 0%). There was a statistically significant difference in effect size between the two subgroups (*p* = 0.009) (Figure 4C). In gait speed, in the subgroup with disease duration ≤3 months, the experimental group performed better than the control group with moderate heterogeneity (MD = 0.18, *p* = 0.0005, *I*^2^ = 43%). In the subgroup with disease duration > 3 months, there was no statistically significant difference between the experimental group and the control group (*p* = 0.06, *I*^2^ = 0%). There was no statistically significant difference in effect size between the two subgroups (*p* = 0.12) (Figure 4D).

#### 3.5.2 Subgroup analyses of training frequency

**Figure 5:**
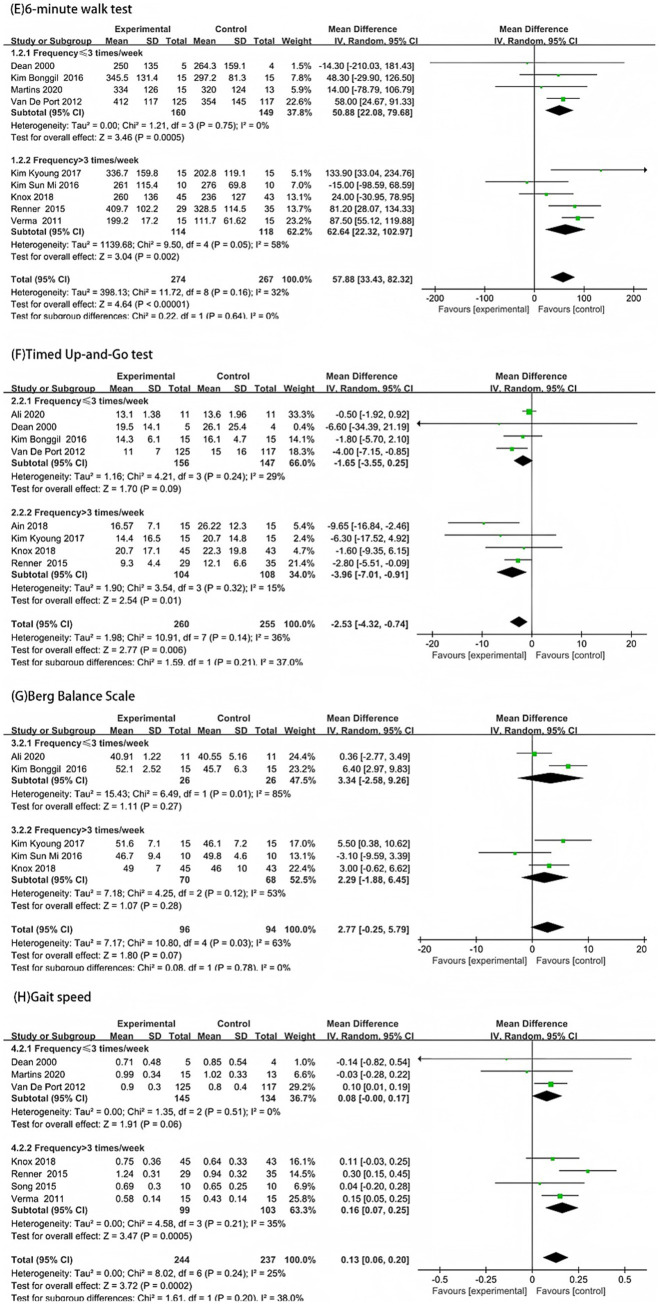
Forest plot subgroup analysis by training frequency: **(E)** 6-Minute Walk Test; **(F)** Timed Up-and-Go Test; **(G)** Berg Balance Scale; and **(H)** Gait Speed.

The subgroup analysis based on training frequency showed that in the 6-min walk test, the experimental groups in both subgroups performed better than the control groups (*p* < 0.05). The high frequency group (>3 times/week) had a higher heterogeneity (MD = 62.64, *I*^2^ = 58%), while the low-frequency group (≤3 times/week) had a lower heterogeneity (MD = 50.88, *I*^2^ = 0%). There was no statistically significant difference in effect size between the two subgroups (*p* = 0.64) (Figure 5E). In the TUG test, there was no statistically significant difference between the experimental and control groups in the low-frequency group (≤3 times/week) (*p* = 0.09, *I*^2^ = 29%), while in the high-frequency group (>3 times/week), the experimental group performed better than the control group (*p* = 0.01, *I*^2^ = 15%). There was no statistically significant difference in effect size between the two subgroups (*p* = 0.21, *I*^2^ = 37%) (Figure 5F).

In the BBS, there was no statistically significant difference between the experimental and control groups in both subgroups (*p* < 0.05). There was no statistically significant difference in effect size between the two subgroups (*p* = 0.78) (Figure 5G). In gait speed, there was no statistically significant difference between the experimental and control groups in the low-frequency group (≤3 times/week) (*p* = 0.06). In the high-frequency group (>3 times/week), the experimental group performed significantly better than the control group (*p* = 0.0005, *I*^2^ = 35%). There was no statistically significant difference in effect size between the two subgroups (*p* = 0.20) (Figure 5H).

#### 3.5.3 Subgroup analyses of single training time

**Figure 6:**
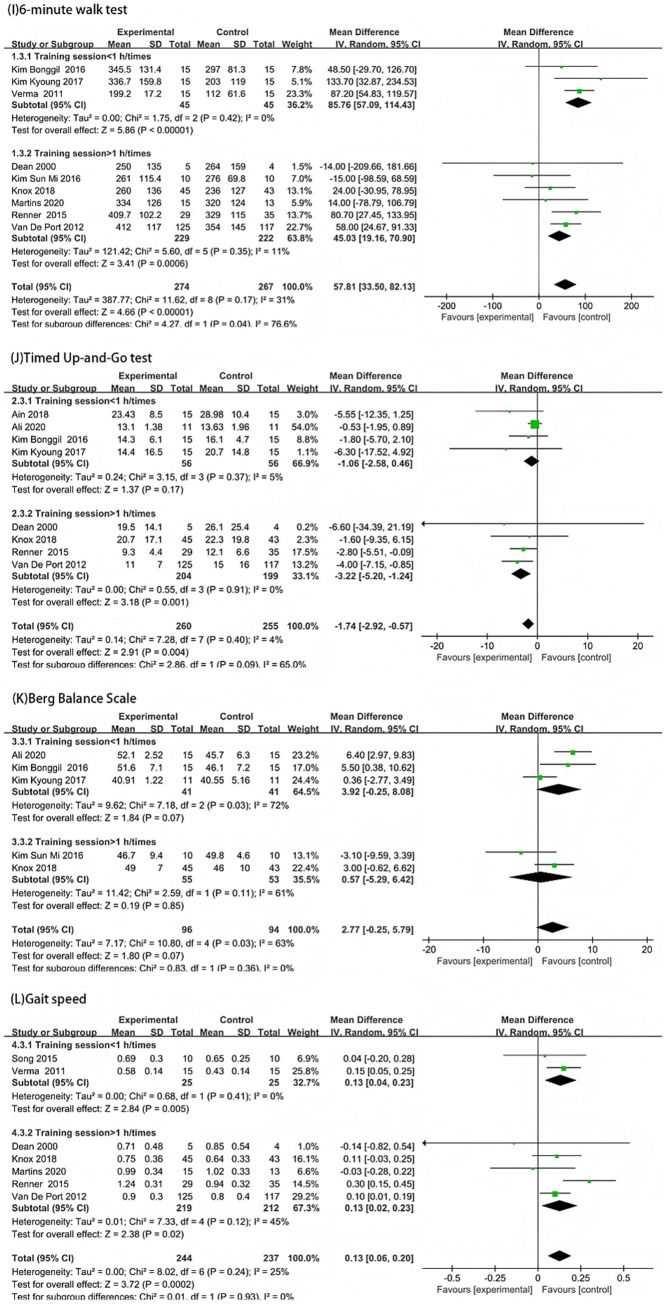
Forest plot subgroup analysis by single-session training duration: **(I)** 6-Minute Walk Test; **(J)** Timed Up-and-Go Test; **(K)** Berg Balance Scale; and **(L)** Gait Speed.

The subgroup analysis based on single training session duration showed that in the 6-min walk test, the experimental groups in both subgroups performed better than the control groups (*p* < 0.05). The heterogeneity was lower in the short-duration training group (<1 h/session) (MD = 85.76, *p* < 0.00001, *I*^2^ = 0%), and lower in the long-duration training group (≥1 h/session) (MD = 45.03, *p* < 0.0006, *I*^2^ = 11%). The difference in effect size between the two subgroups was statistically significant (*p* = 0.04) (Figure 6I). In the TUG test, the difference between the experimental and control groups was not significant in the short-duration training group (<1 h/session) (*p* = 0.17, *I*^2^ = 5%). In the long-duration training group (≥1 h/session), the difference was significant (MD = −3.22, *p* = 0.001, *I*^2^ = 0%). The difference in effect size between the two subgroups was not statistically significant (*p* = 0.09) (Figure 6J).

In the Berg BBS, there was no statistically significant difference between the experimental and control groups in both subgroups (*p* > 0.05), and the difference in effect size between the two subgroups was also not statistically significant (*p* = 0.36) (6 K). In gait speed, the experimental groups in both subgroups performed better than the control groups (*p* < 0.05). The heterogeneity was lower in the short-duration training group (<1 h/session) (MD = 0.13, *p* = 0.005, *I*^2^ = 0%), and lower in the long-duration training group (≥1 h/session) (MD = 0.13, *p* = 0.02, *I*^2^ = 45%). The difference in effect size between the two subgroups was not statistically significant (*p* = 0.93) (Figure 6L).

#### 3.5.4 Subgroup analyses of different duration of follow-up

**Figure 7:**
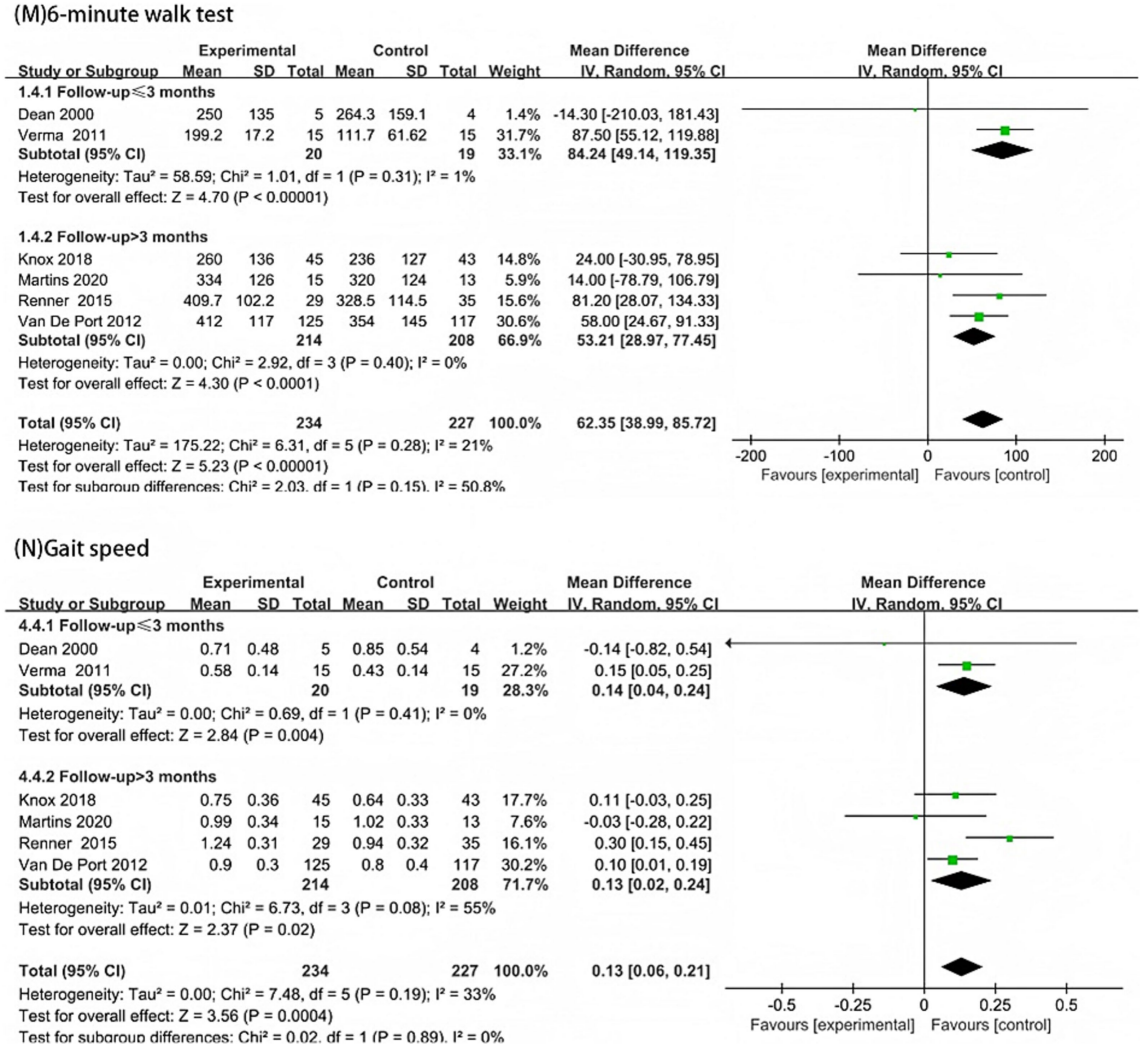
Forest plot subgroup analysis by follow-up duration: **(M)** 6-Minute Walk Test; and **(N)** Gait Speed.

The subgroup analysis based on follow-up duration showed that in the 6-min walk test, the experimental group performed better than the control group (*p* < 0.05), and the heterogeneity of both subgroups was relatively low (*I*^2^ < 50%). There was no statistically significant difference in effect size between the two subgroups (*p* = 0.15) (Figure 7M). About gait speed, the experimental group performed better than the control group (*p* < 0.05). The heterogeneity was low in the group with a follow-up time of ≤3 months (*I*^2^ = 0%), while it was high in the group with a follow-up time of > 3 months (*I*^2^ = 55%). There was no statistically significant difference in the effect size between the two subgroups (*p* = 0.89) (Figure 7N).

### 3.6 Sensitivity analysis and publication bias

**Figure 8:**
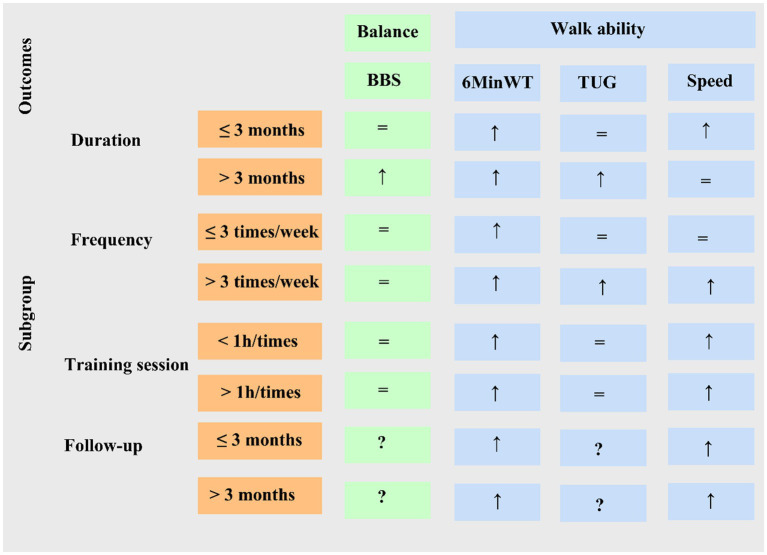
Summary of found evidence for subgroups. (↑) Beneficial effect, *p* < 0.05; (=) the same effect, *p* > 0.05; (?) unknown effect. BBS, Berg Balance Scale; 6MinWT, 6-min walk test; TUG, Timed Up-and-Go test.

**Figure 9:**
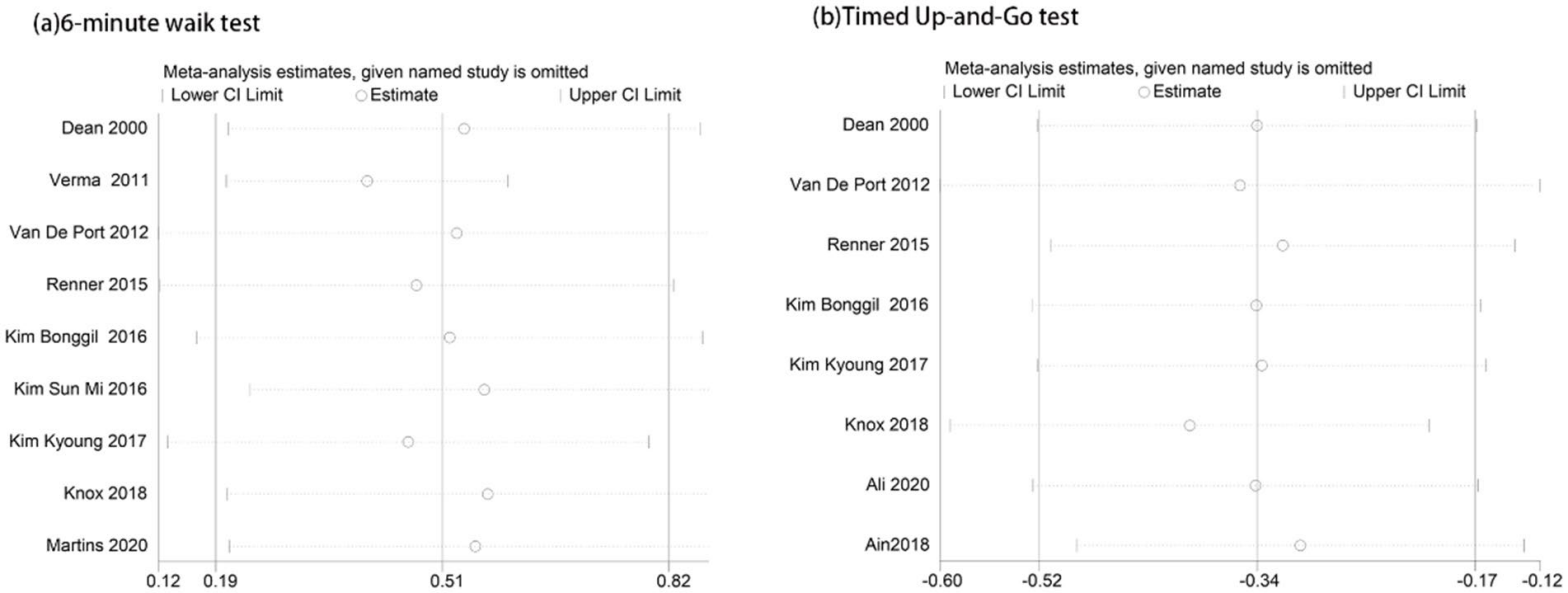
Sensitivity analysis results: **(a)** 6-Minute Walk Test; and **(b)** Timed Up-and-Go Test (Appendix 3).

**Figure 10:**
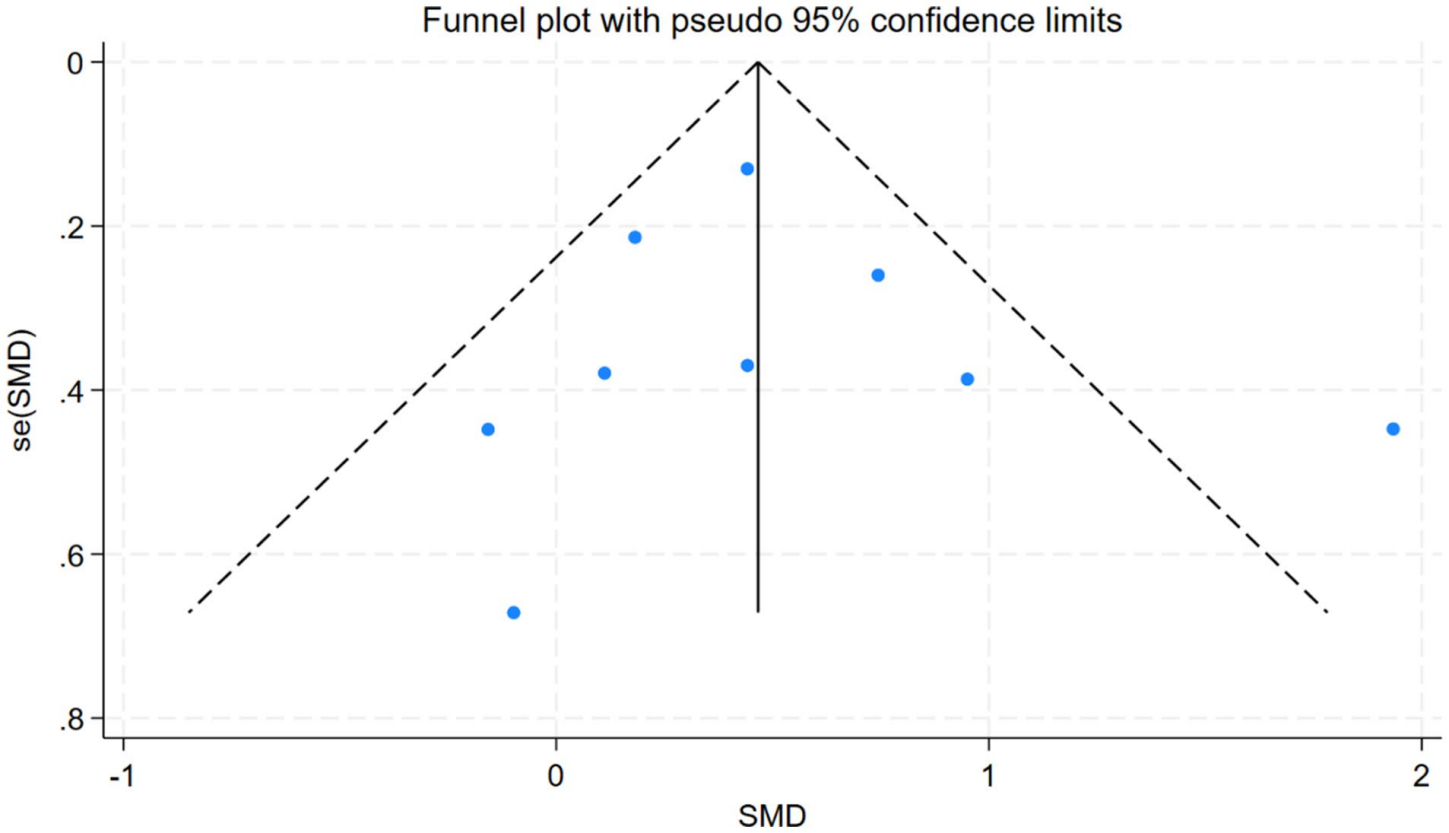
Funnel plot of publication bias for 6-minute walk test score (Appendix 3).

Subgroup analysis results are summarized (Figure 8). Due to the limited number of studies for each intervention, sensitiv¬ity analysis was performed only for the 6-min walk test and the TUG test to explore the stability of the meta-analysis, results were stable (Figure 9). Evaluation of publication bias for studies including 6-min walk test scores showed a funnel plot with near symmetry. The Egger test (p = 0.721 > 0.05) showed no significant publication bias (Figure 10).

## 4 Discussion

This meta-analysis demonstrates that task-oriented CCT significantly increases the distance covered during the 6-min walk test, confirming its efficacy in improving walking endurance. The reduced time required to complete the TUG indicates enhanced functional mobility. In stroke patients with lower limb dysfunction, task-oriented CCT leads to significantly greater improvements in walking speed compared to conventional training, although no significant differences were observed in BBS or MBI scores.

Subgroup analysis further revealed that, compared with early intervention within 3 months, delivering task-oriented CCT to individuals with a disease duration exceeding 3 months—particularly at a higher training frequency (≥3 times/week) and with individual sessions lasting ≤60 min—resulted in more pronounced improvements in walking endurance, as measured by the 6-min walk test. These benefits were most evident during the 3-month follow-up period and persisted over the long term, albeit with a gradually diminishing rate of improvement. Higher training frequency (>3 times/week) or longer session duration (≥1 h) was associated with greater gains in mobility (TUG), especially among patients with prolonged post-stroke duration (>3 months). For balance (BBS), patients with a longer post-stroke interval (>3 months) showed greater responsiveness, whereas neither training frequency nor session duration had a significant influence. Improvements in walking speed were primarily driven by higher training frequency (≥3 times/week) and were more substantial in individuals with a shorter disease duration (≤3 months).

For stroke patients, balance is often impaired, resulting in increased postural sway amplitudes and altered weight distribution patterns (31–33). Dynamic balance plays a critical role in mobility, and balance disorders are closely associated with an elevated risk of falls among stroke survivors (34–37). Although many stroke patients may regain the ability to walk, their walking endurance is frequently limited, even in those with relatively high functional capacity. Both dynamic balance and walking endurance are significantly correlated with health-related quality of life and participation levels in stroke survivors (38). Riech et al. (39) conducted a systematic review of training methods for standing and walking in adult stroke patients and first recommended repetitive task-specific interventions delivered in a group setting, as this approach demonstrated the largest statistically significant and clinically meaningful improvements in walking ability.

Task-oriented CCT simulates real-life environments through repetitive tasks, such as obstacle crossing and turning exercises, to enhance sensory-motor integration and cortical reorganization, thereby promoting neuroplasticity. It addresses multiple components of gait rehabilitation within a single session, including strength training, balance improvement, and steady-state walking practice. Task-oriented CCT incorporates at least three key principles essential for effective and efficient rehabilitation: First, exercises conducted at different stations allow stroke patients to engage in progressive, individualized programs tailored to their specific needs (10). Second, each therapist can supervise 2–3 patients simultaneously, reducing dependence on one-on-one therapist support (33, 40) and enabling increased treatment intensity and repetition without requiring additional human resources. Third, task-oriented CCT is delivered in a group setting, which facilitates observational learning—where participants observe others relearning motor tasks—and encourages social interaction among patients. Circuit training programs have been shown not only to improve motor function in stroke survivors (30, 41–43) but also to benefit cardiovascular fitness, muscle strength, and endurance and may exert a positive effect on post-stroke depression (44). Stroke patients can enhance their physical performance by integrating task-specific exercises into structured circuit training protocols (9, 24, 45). The Dutch Physical Therapy Association (KNGF) guidelines recommend this training method, as it has been demonstrated to enhance walking distance and speed, walking ability, sitting balance, and general health (Grade 1) (46).

McEwen et al. (47) analyzed 16 RCTs on cyclic exercise protocols for post-stroke survivors. However, due to generally poor reporting quality, an optimal standardized cyclic training protocol for post-stroke rehabilitation could not be identified. While individualized training protocols remain crucial for addressing the diverse needs of stroke survivors, there is a clear need to develop a unified foundational protocol to support broader implementation and consistency across clinical settings. Such a standardized protocol should specify multiple training stations, the type of exercise performed at each station, the duration and intensity of each exercise component, as well as the specific functional or physiological goals expected.

Four systematic reviews and one Cochrane review indicate that increasing the intensity and/or duration of exercise therapy can lead to small to moderate improvements in activities of daily living, walking ability, and walking speed (8, 11, 48, 49). Research has identified a clear dose–response relationship, where higher treatment doses are associated with clinically meaningful gains (12, 50). It is essential to individualize training intensity and duration according to specific rehabilitation goals. This study has several limitations: First, the relatively small number of included studies limits the generalizability of the findings. Second, the included studies exhibit clinical heterogeneity in terms of participant characteristics (including stroke type and stage), intervention protocols (such as dosage, intensity, and frequency), and outcome measures related to balance. This variability may affect the validity of pooled results in meta-analyses, thereby influencing the robustness of the conclusions. Third, only English-language literature was searched, introducing language bias and potentially excluding relevant non-English studies. Fourth, only five representative outcome indicators were analyzed, with limited consideration of emotional or psychological factors in patients, potentially leading to an incomplete understanding of the intervention’s overall impact. Fifth, some studies have small sample sizes, inadequate descriptions of interventions that hinder replication, and high heterogeneity among outcome indicators, which prevent meaningful statistical pooling through meta-analysis. Sixth, the Modified Barthel Index was included as an outcome measure; however, MBI is not specific to lower limb function and also encompasses assessments of upper limb function and activities of daily living. Finally, many studies lack long-term follow-up data, making it difficult to assess the sustained effects of group-based training on lower limb functional impairments in stroke survivors.

This meta-analysis suggests that task-oriented CCT may offer potential benefits in improving lower limb function following a stroke compared with traditional interventions. However, there is currently a paucity of studies evaluating the long-term health economic outcomes associated with task-oriented CCT versus traditional one-on-one treatment. Furthermore, future large-scale, multicenter, RCTs are necessary to compare the effects of various training components—such as content, intensity, session duration, and timing of intervention—on post-stroke walking function. Additionally, the potential synergistic effects of integrating task-oriented CCT with emerging technologies, such as robot-assisted therapy or brain-computer interface systems, warrant further investigation.

## Data Availability

The original contributions presented in the study are included in the article/Supplementary material, further inquiries can be directed to the corresponding author.
